# Coronary artery stenosis, plaque burden, and severity of myocardial ischemia

**DOI:** 10.1093/ehjimp/qyaf139

**Published:** 2025-12-11

**Authors:** Tanja Kero, Juhani Knuuti, Sarah Bär, Jeroen J Bax, Antti Saraste, Teemu Maaniitty

**Affiliations:** Nuclear Medicine and PET, Department of Surgical Sciences, Uppsala University, Uppsala, Sweden; PET Center/Medical Imaging Center, Uppsala University Hospital, 75185 Uppsala, Sweden; Turku PET Centre, Turku University Hospital and University of Turku, Turku, Finland; Turku PET Centre, Turku University Hospital and University of Turku, Turku, Finland; InFlames Flagship, University of Turku, Turku, Finland; Department of Clinical Physiology, Nuclear Medicine, and PET, Turku University Hospital, Turku, Finland; Turku PET Centre, Turku University Hospital and University of Turku, Turku, Finland; Department of Cardiology, Bern University Hospital Inselspital, Bern, Switzerland; Department of Cardiology, Leiden University Medical Center, Leiden, The Netherlands; Turku PET Centre, Turku University Hospital and University of Turku, Turku, Finland; Heart Center, Turku University Hospital, University of Turku, Turku, Finland; Turku PET Centre, Turku University Hospital and University of Turku, Turku, Finland; Department of Clinical Physiology, Nuclear Medicine, and PET, Turku University Hospital, Turku, Finland

**Keywords:** coronary computed tomography angiography, artificial intelligence, coronary plaque, ischaemia, positron emission tomography

## Abstract

**Aims:**

The relationship between the extent and composition of coronary atherosclerosis and the severity of myocardial ischaemia remains incompletely understood. We assessed whether artificial intelligence-guided coronary computed tomography angiography–derived plaque burden and composition correlate with ischaemia severity.

**Methods and results:**

We included 837 symptomatic patients undergoing coronary computed tomography angiography and subsequent ^15^O-water positron emission tomography myocardial perfusion imaging. Artificial intelligence–guided coronary computed tomography angiography was used to quantify plaque features—diameter stenosis, percent atheroma volume (PAV), percent non-calcified plaque volume (NCPV), and percent calcified plaque volume (CPV)—per patient and per major coronary artery (LAD, LCx, RCA). Ischaemia severity was classified into four categories based on regional hyperaemic myocardial blood flow. Increasing severity of ischaemia was associated with higher diameter stenosis and plaque burden (PAV, NCPV, CPV) on patient level and in all major coronary territories (overall *P* < 0.001). The LAD consistently demonstrated higher atherosclerotic burden as compared to the LCx and RCA. Ordinal logistic regression confirmed that diameter stenosis (OR 1.02–1.03, *P* < 0.001) and NCPV (OR 1.04–1.05, *P* = 0.011–0.031) were significant predictors of ischaemia severity in all coronary arteries, while CPV was predictive only in the LAD and RCA (OR 1.03–1.04, *P* = 0.002–0.015).

**Conclusion:**

Artificial intelligence–guided coronary computed tomography angiography–derived measures of plaque burden and stenosis are associated with the severity of myocardial ischaemia, although overlapping distributions across ischaemia severity indicate that anatomical imaging alone may be insufficient for accurate phenotyping of flow-limiting CAD. These findings encourage for the integration of functional imaging with quantitative plaque analysis for a more comprehensive evaluation of coronary artery disease.

## Graphical Abstract

**Graphical Abstract:**
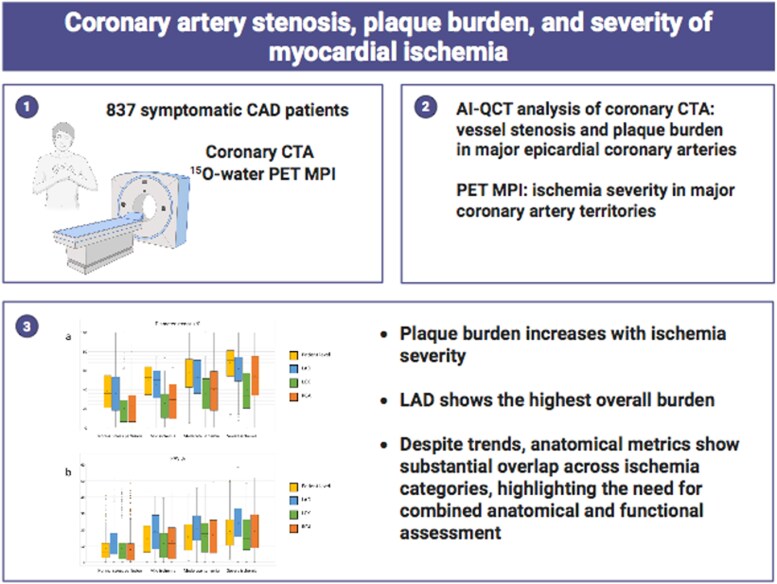
**Relationship Between Coronary Plaque Burden and Ischemia Severity.** This graphical abstract illustrates the association between quantitative coronary plaque characteristics and severity of myocardial ischemia. AI-guided coronary computed tomography angiography (AI-QCT) was used to assess plaque burden—measured as diameter stenosis, percent atheroma volume (PAV), non-calcified plaque volume (NCPV), and calcified plaque volume (CPV)—at patient level and in the three major coronary arteries: left anterior descending (LAD), left circumflex (LCX), and right coronary artery (RCA). As shown, increasing stenosis (a) and PAV (b) generally correspond with increasing ischemia severity, categorized by PET-derived myocardial blood flow into normal, mild, moderate, and severe ischemia. Notably, despite overall trends, substantial overlap in plaque metrics across ischemia categories suggests that anatomical assessment alone may not fully explain functional impairment, supporting the integration of anatomical and functional imaging for comprehensive coronary artery disease evaluation. Created in BioRender. kero, t. (2025) https://BioRender.com/knx9oj2.

## Introduction

Coronary computed tomography angiography (CTA) has over the recent years become the first-line test for suspected coronary artery disease (CAD) in patients with a moderate or low pre-test clinical likelihood of CAD.^1^ Recently, application of artificial intelligence to the analysis of coronary CTA has enabled rapid, objective, and reproducible quantification of coronary artery stenosis and plaque volumes and composition such as calcified, non-calcified, and low-density fractions.^2–4^ Artificial intelligence–guided quantitative computed tomography (AI-QCT) is a novel, FDA-approved coronary stenosis and plaque characterization and quantification tool.^2,3^

Atherosclerotic plaque quantity and composition and high-risk plaque characteristics are known to have diagnostic and prognostic value.^5^ Emerging evidence also suggests a relationship between various plaque features and presence of myocardial ischaemia in patients with CAD.^6–8^ Plaque burden and phenotype appears also to differ between the major coronary arteries.^9^

In recent studies, we found that both AI-QCT measures of luminal narrowing and plaque burden are independent predictors of the existence of myocardial ischaemia defined by positron emission tomography (PET) myocardial perfusion imaging (MPI), both at per-patient level^10^ and per-vessel level.^11^ Surprisingly, many vessels supplying ischaemic regions had relatively low stenosis degree and plaque burden, especially in LCx and RCA vessels.^11^ Our hypothesis was that not only the presence but also the severity myocardial ischaemia is associated with coronary stenosis and plaque burden. In addition, we hypothesized that this relationship might differ between the coronary arteries. Therefore, in this study, we assessed and compared the atherosclerotic plaque burden and plaque phenotype of the major epicardial coronary arteries supplying myocardial territories with various degrees of ischaemia.

## Methods

### Patients

The study cohort was derived from the Turku Cardiac CTA Registry at Turku University Hospital, Finland. The registry includes consecutive symptomatic patients who underwent coronary CTA for suspected CAD between February 2007 and December 2016. Patients with previously known obstructive CAD were not considered for inclusion.

According to the local imaging protocol, patients with suspected CAD first undergo coronary CTA. If CTA reveals at least one suspected obstructive coronary artery stenosis (defined as a visually estimated ≥50% diameter reduction), the patient is referred for downstream stress ^15^O-water PET MPI to assess the haemodynamic significance of the stenosis. For patients without visually obstructive stenosis, no further imaging is required. Demographic data, cardiovascular risk factors, and symptoms were retrospectively collected from the medical records at Turku University Hospital.

The study complies with the Declaration of Helsinki. The Ethics Committee of the Hospital District of Southwest Finland approved the study protocol and waived the requirement for written informed consent.

### Coronary CTA and PET imaging procedures

Coronary CTA scans were performed with 64-row hybrid PET-CT scanners (GE Discovery VCT or GE D690, General Electric Medical Systems, Waukesha, USA) as previously described.^12^ Intravenous metoprolol (0–30 mg) and oral/sublingual nitrate were administered before the CTA imaging. Coronary CTA was performed using intravenously administered low-osmolar iodine contrast agent. Prospectively triggered acquisition was applied whenever feasible.

Based on the initial visual evaluation of the coronary CTA images, patients with suspected obstructive stenosis on CTA underwent dynamic quantitative PET perfusion scan with ^15^O-labeled water [(^15^O)H_2_O] during adenosine vasodilator infusion (140 µg/kg/min) using a hybrid PET-CT scanner, usually in the same imaging session with CTA.^12,13^ The patients were instructed to abstain from caffeine for 24 h before the PET study.

### AI-QCT analysis

Coronary CTA scans were re-analyzed in 2022–2023 in a blinded manner using a previously described AI-QCT algorithm (Cleerly LABS, Cleerly Inc., Denver, CO, USA).^2,3^ This commercially available, FDA-approved software utilizes a series of validated convolutional neural networks (3D U-Net and VGG network variants) for image quality assessment, coronary artery segmentation and labelling, lumen and wall evaluation, vessel contour determination, and plaque characterization.

The AI-QCT algorithm allows for assessing coronary artery lesions where plaque is present. Using a standard cross-sectional slice of the normal proximal reference vessel, along with slices at the start and end of the lesion and the slice showing the most severe narrowing, the percentage diameter stenosis was automatically calculated.

The remodeling index (RI) is defined as the ratio of the outer vessel diameter to the mean diameter of the normal adjoining segments, with positive remodelling defined as RI ≥1.1.^14^

Within coronary artery lesions, plaque volume was quantified and further characterized as low-attenuation plaque, non-calcified plaque, or calcified plaque based upon Hounsfield unit (HU) densities of <30, 30 to 350, and >350, respectively.^2^ Vessel volumes, lumen volumes, plaque volumes, diameter and area stenosis, and RI were recorded for each major coronary artery including their side branches (>1.5 mm in diameter). Plaque volumes (mm^3^) were calculated for each coronary lesion and then summed to compute the total plaque volumes in the left main (LM), left anterior descending (LAD), left circumflex (LCx), and right coronary artery (RCA); variables for the LM artery were subsequently integrated with the LAD and LCx. The total plaque burden of each vessel was calculated as total plaque volume divided by the vessel volume, expressed as percent atheroma volume (PAV) (%). Similarly, calcified and non-calcified plaque volumes were divided by the vessel volume to obtain percent calcified plaque volume (CPV) (%) and percent non-calcified plaque volume (NCPV) (%), respectively. Due to small quantities, the absolute number of low attenuation plaque volume (LAPV) was incorporated into NCPV, and the presence or absence of any low-attenuation plaque was reported as a binary variable. The most severe degree of stenosis and the highest RI were reported for each major coronary artery.

### PET analysis

The dynamic PET perfusion data were quantitatively analyzed using Carimas software (developed at the Turku PET Centre, Turku, Finland). Hyperaemic myocardial blood flow (MBF), expressed in mL min^−1^g^−1^ of perfusable myocardial tissue, was calculated for the standardized 17 segments, in accordance with the American Heart Association guidelines.^15^ Individual myocardial segments were assigned to the three major coronary arteries as recommended. Reduced hyperaemic MBF was taken as a marker of inducible ischaemia (i.e. impaired stress perfusion with potential ischaemic consequences) in this patient cohort with suspected, but not previously known CAD. Myocardial ischaemia was thus defined as hyperaemic MBF ≤ 2.30 mL min^−1^g^−1^ in at least two adjacent segments, excluding the basal septum.^16^ Regional hyperaemic MBF was calculated as the mean of two adjacent segments with the lowest perfusion within a coronary artery territory (vessel-level analysis) or across the whole left ventricular myocardium (patient-level analysis). The severity of myocardial ischaemia was categorized into four groups based on the regional hyperaemic MBF:

Normal perfusion: hyperaemic MBF >2.30 mL × min^−1^g^−1^

Mild ischaemia: hyperaemic MBF 2.00–2.30 mL × min^−1^g^−1^

Moderate ischaemia: hyperaemic MBF 1.50–2.00 mL × min^−1^g^−1^

Severe ischaemia: hyperaemic MBF <1.50 mL × min^−1^g^−1^.

Vessel dominance was defined based on the origin of the posterior descending artery (PDA) on coronary CTA; in right-dominant anatomy, the PDA originates from the right coronary artery (RCA); in left-dominant anatomy, from the left circumflex artery (LCx); and in co-dominant anatomy, from both the RCA and LCx. In right-dominant and co-dominant cases, the inferoseptal and inferior myocardial segments were assumed to be perfused by the RCA and assigned to the RCA myocardial territory. In left-dominant cases, these segments were assumed to be perfused by the LCx and were incorporated into the LCx myocardial territory.

### Statistical analysis

Continuous variables are presented as mean ± standard deviation (SD) or median [interquartile range (25th–75th percentile)], as appropriate. Categorical variables are presented as counts with percentages. The Shapiro–Wilk test was used to assess the normality of the data distribution.

Different coronary arteries (LAD, LCx, RCA) were considered as independent samples. The independent-samples Kruskal–Wallis test was used to compare continuous variables and the Chi-square test was used for categorical variables. For pairwise comparisons, significance values were adjusted by the Bonferroni correction for multiple testing or Dunn’s multiple comparisons test.

Multivariable ordinal logistic regression was employed to investigate the relationship between the severity of myocardial ischaemia (measured on an ordinal scale) and AI-QCT-derived atherosclerosis variables, while adjusting for degree of stenosis (entered as a continuous variable) and other relevant covariates. We evaluated multicollinearity using the variance inflation factor (VIF), and variable combinations were selected accordingly to ensure independent contributions. Two ordinal logistic regression models were used with the following predictors:

Model 1: diameter stenosis, percent atheroma volume (PAV), presence of low attenuation plaque, and RI.

Model 2: diameter stenosis, percent non-calcified plaque volume (NCPV), percent calcified plaque volume (CPV), presence of low attenuation plaque, and RI.

Analyses were two-tailed and a *P*-value <0.05 was considered statistically significant. Statistical analyses were performed using IBM SPSS statistics for Macintosh (Version 29.0.2.0 Armonk, NY: IBM Corp) and GraphPad Prism (version 10.5.0, GraphPad Software, San Diego, California, USA).

## Results

### Study cohort

**Figure 1:**
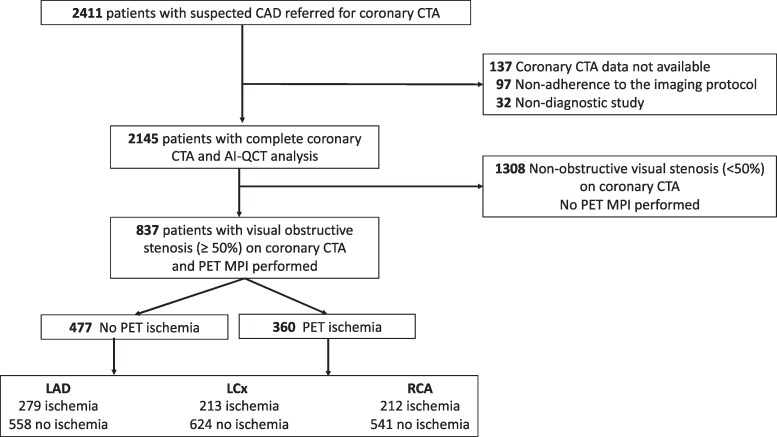
Patient flowchart. AI-QCT, artificial intelligence-guided quantitative computed tomography; CAD, coronary artery disease; CTA, computed tomography angiography; MPI, myocardial perfusion imaging; LAD, left anterior descending coronary artery; LCx, left circumflex coronary artery; PET, positron emission tomography; RCA, right coronary artery. This figure was published in *J Nucl Cardiol*. Nov;53:102470. doi: 10.1016/j.nuclcard.2025.102470. Epub 2025 Aug 9. PMID: 40789366. Kero T, Knuuti J, Bär S, Bax JJ, Saraste A, Maaniitty T: “Stenosis degree and plaque burden differ between the major epicardial coronary arteries supplying ischemic territories”. Copyright Elsevier (2025).

Of the 2411 consecutive symptomatic patients with coronary CTA, 837 (35%) patients proceeded to downstream PET MPI according to the local selective hybrid imaging protocol and had their coronary CTA quantitatively analysed by AI-QCT (*Figure 1*). Among these 837 patients, 360 (43%) demonstrated myocardial ischaemia based on the presence of abnormal regional stress perfusion on a patient level.

Right coronary dominance was present in 703 patients (84%), left dominance in 84 patients (10%), and co-dominance in 50 patients (6%). At the vessel-level, ischaemia was present in 279 (33%) of the LAD territories, 213 (25%) of the LCx territories, and 212 (28%) of the RCA territories (*P* < 0.001). Among ischaemic myocardial territories, regional hyperaemic MBF median (interquartile range) was 1.61 (1.19–1.90) mL × min^−1^g^−1^ in the LAD, 1.65 (1.26–1.93) mL × min^−1^g^−1^ in the LCx and 1.50 (1.13–1.79) mL × min^−1^g^−1^ in the RCA territories (*P* < 0.001).

The clinical characteristics of the patients are shown in *Table 1*.

**Table 1:** Patient characteristics

Patient characteristics	*n* = 837
Age, years	65 (59–71)
Sex (male), *n* (%)	441 (52.7%)
Hypertension, *n* (%)	552 (65.9%)
Dyslipidemia, *n* (%)	594 (71.0%)
Current/previous smoker, *n* (%)	328 (39.2%)
Diabetes mellitus, *n* (%)	156 (18.6%)
Family history of CAD, *n* (%)	394 (47.1%)
Typical angina, *n* (%)	243 (29.0%)
BMI, kg/m^2^	27.3 (24.7–30.5)
Agatston coronary calcium score	248.5 (43.0–671.3)
**Medication**	
Antiplatelet drug, *n* (%)	445 (53.2%)
Lipid-lowering drug, *n* (%)	421 (50.3%)
Betablocker, *n* (%)	438 (52.3%)
Long-acting nitrate, *n* (%)	92 (11.0%)
Calcium channel blocker, *n* (%)	166 (19.8%)
ACE inhibitor, *n* (%)	175 (20.9%)
AT II antagonist, *n* (%)	197 (23.5%)

Values are *n* (%) or median (interquartile range).

ACE, angiotensin-converting enzyme; AT II, angiotensin II; BMI, body mass index.

### Stenosis degree and plaque features according to the severity of ischaemia

**Figure 2:**
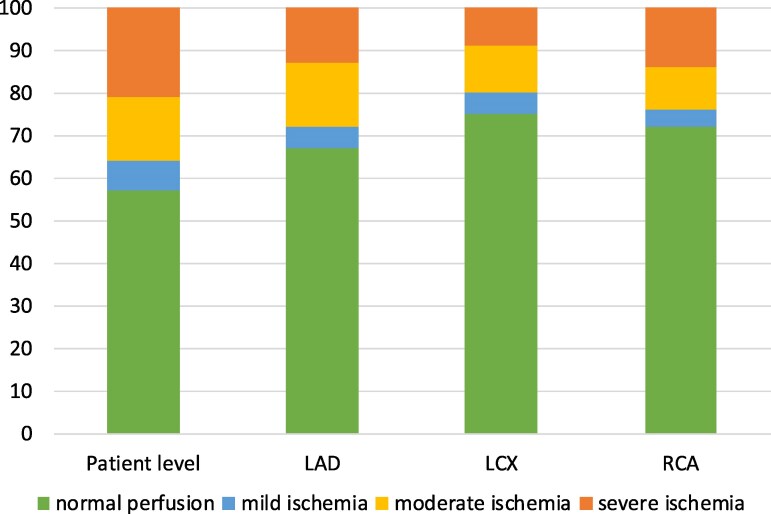
The distribution (%) of normal perfusion and mild, moderate, and severe ischaemia at patient level and in the main coronary artery regions.

The distribution of normal perfusion and mild, moderate, and severe ischaemia at a per patient level and in the major coronary artery territories is shown in *Table 2* and in *Figure 2*. There was an overall difference in distribution of ischaemia severity between vessels (*P* = 0.001).

**Table 2:** The distribution, ***n* (%), of perfusion findings at per patient and per territory levels**

	Patient level	LAD	LCX	RCA
Normal perfusion	477 (57%)	558 (67%)	624 (75%)	541 (72%)
Mild ischaemia	60 (7%)	45 (5%)	40 (5%)	28 (4%)
Moderate ischaemia	125 (15%)	125 (15%)	92 (11%)	77 (10%)
Severe ischaemia	175 (21%)	109 (13%)	81 (10%)	107 (14%)

**Figure 3:**
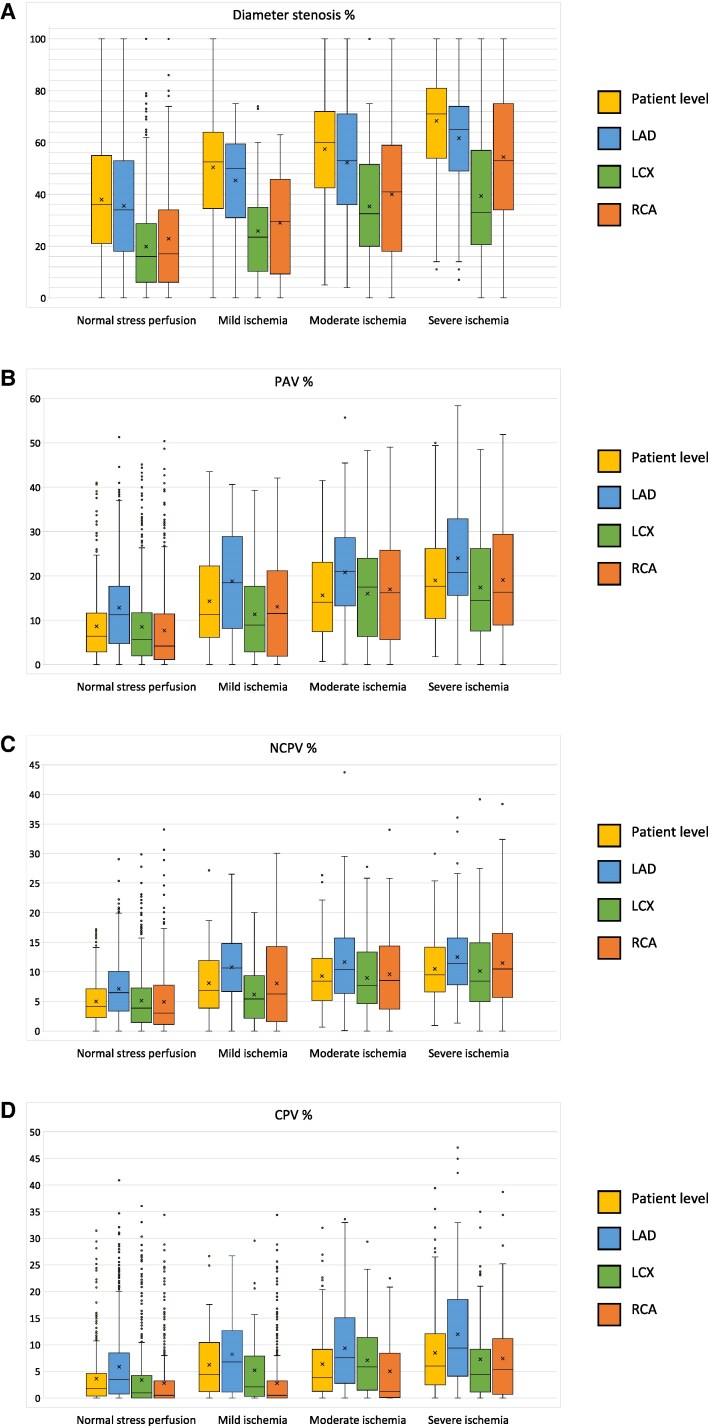
Boxplots showing the distribution of diameter stenosis (*A*), PAV (*B*), NCPV (*C*), and CPV (*D*) at per patient level and vessel level for the main coronary arteries supplying myocardial territories with normal perfusion and mild, moderate and severe ischaemia. The boxes represent the interquartile range (IQR) and the median, the whiskers show the largest and smallest values within 1.5 × IQR, dots represent outliers, and the X in the box represents mean value.

*Table 3* and *Figure 3* illustrate the distribution of coronary atherosclerotic metrics; diameter stenosis, PAV, percent non-calcified plaque volume (NCPV), and percent CPV, both at patient level and stratified by main coronary artery. Across all four measures, the LAD consistently exhibited higher atherosclerotic burden than the LCx and the RCA.

**Table 3:** The diameter stenosis, PAV, NCPV, and CPV at per patient level and in the vascular territories with normal perfusion or mild, moderate, or severe ischaemia

	Normal	Mild ischaemia	*P*-value (normal-mild)	Moderate ischaemia	*P*-value (mild-moderate)	Severe ischaemia	*P*-value (moderate-severe)	*P*-value (overall)
**Diameter stenosis %**								
Patient level	36 (21–55)	53 (35–64)	0.001	60 (43–72)	0.428	71 (54–81)	0.004	<0.001
LAD	34 (18–53)^a,b^	50 (31–60)^a,b^	0.028	53 (36–71)^a,b^	0.853	65 (49–74)^a^	0.022	<0.001
LCX	16 (6–29)^a^	24 (10–35)^a^	0.196	33 (20–52)^a^	0.170	33 (21–57)^a,c^	1.000	<0.001
RCA	17 (6–34)^b^	30 (9–46)^b^	0.604	41 (18–59)^b^	0.555	53 (34–75)^c^	0.061	<0.001
*P-*value (overall between vessels)	<0.001	<0.001		<0.001		<0.001		
**PAV %**								
Patient level	6.4 (2.9–11.7)	11.3 (6.2–22.3)	<0.001	14.4 (7.5–23.1)	1.000	17.7 (10.4–26.2)	0.078	<0.001
LAD	11.3 (4.8–17.7)^a,b^	18.5 (8.2–28.9)^a^	0.005	21.0 (13.2–28.6)^a,b^	1.000	20.8 (15.7–32.9)^a,b^	0.716	<0.001
LCX	5.7 (1.9–11.7)^a^	8.9 (2.8–17.7)^a^	0.350	17.5 (6.4–24.0)^a^	0.111	14.5 (7.6–26.2)^a^	1.000	<0.001
RCA	4.2 (1.2–11.4)^b^	11.6 (1.9–21.2)	0.119	16.2 (5.6–25.8)^b^	0.821	16.3 (8.9–29.4)^b^	1.000	<0.001
*P-*value (overall between vessels)	<0.001	0.009		0.005		<0.001		
**NCPV %**								
Patient level	4.1 (2.2–7.1)	6.9 (3.9–11.9)	<0.001	8.4 (5.1–12.3)	0.983	9.4 (6.6–14.2)	0.217	<0.001
LAD	6.5 (3.3–10.1)^a,b^	10.6 (6.7–14.8)^a^	<0.001	10.4 (6.4–15.7)^a^	1.000	11.3 (7.8–15.7)^a^	1.000	<0.001
LCX	3.9 (1.4–7.3)^a^	5.4 (2.2–9.3)^a^	0.746	7.6 (4.6–13.3)^a^	0.093	8.4 (5.0–14.8)^a^	1.000	<0.001
RCA	3.0 (1.1–7.8)^b^	6.3 (1.6–14.3)	0.179	8.5 (3.7–14.4)	0.872	10.5 (5.7–16.5)	1.000	<0.001
*P-*value (overall between vessels)	<0.001	0.003		0.01		0.033		
**CPV %**								
Patient level	1.8 (0.4–4.6)	4.4 (1.2–10.4)	0.001	3.8 (1.3–9.2)	1.000	6.0 (2.5–12.1)	0.085	<0.001
LAD	3.5 (0.7–8.5)^a,b^	6.8 (1.1–12.6)^b^	0.238	7.5 (2.7–15.1)^b^	1.000	9.4 (4.1–18.5)^a,b^	1.000	<0.001
LCX	0.9 (0.0–4.2)^a,c^	2.1 (0.3–7.9)^c^	0.105	5.8 (1.5–11.4)	0.548	4.4 (1.1–9.2)^a^	1.000	<0.001
RCA	0.5 (0.0–3.2)^b,c^	1.2 (0.1–8.4)^b,c^	0.346	5.4 (0.7–11.1)^b^	1.000	3.6 (1.1–11.0)^b^	1.000	<0.001
*P-*value (overall between vessels)	<0.001	<0.001		0.001		<0.001		
**Low attenuation plaque present (*n*)%**								
Patient level	134 (28%)	28 (47%)	0.009	68 (54%)	0.973	105 (60%)	1.000	<0.001
LAD	120 (22%)^a,b^	19 (42%)^a^	0.005	53 (42%)^a^	1.000	53 (49%)^a^	1.000	<0.001
LCX	79 (13%)^a^	6 (15%)^a^	1.000	16 (17%)^a^	1.000	23 (28%)^a^	0.252	0.002
RCA	76 (14%)^b^	10 (36%)	0.005	21 (27%)	1.000	42 (39%)	0.273	<0.001
*P-*value (overall between vessels)	<0.001	0.021		<0.001			0.019	
**Remodeling index**								
Patient level	1.4 (1.2–1.5)	1.5 (1.4–1.6)	<0.001	1.5 (1.4–1.6)	1.000	1.5 (1.4–1.7)	0.119	<0.001
LAD	1.3 (1.2–1.4)^a,b^	1.4 (1.2–1.5)	0.026	1.4 (1.3–1.5)^a,b^	1.000	1.5 (1.3–1.6)	0.503	<0.001
LCX	1.2 (1.1–1.4)^a^	1.3 (1.2–1.4)	0.639	1.3 (1.2–1.4)^a^	1.000	1.4 (1.2–1.6)	1.000	<0.001
RCA	1.2 (1.1–1.4)^b^	1.3 (1.2–1.4)	0.071	1.3 (1.2–1.4)^b^	1.000	1.4 (1.2–1.6)	1.000	<0.001
*P-*value (overall between vessels)	<0.001	0.123		0.001		0.082		

Values are median (interquartile range) or *n* (%).

CPV, percent calcified plaque volume; NCPV, percent non-calcified plaque volume; PAV, percent atheroma volume.

^a,b,c^Denotes significant difference (*P* < 0.05) comparing variables between LAD and LCx (a), between LAD and RCA (b) and between LCx and RCA (c).

On patient level and in all main coronary arteries, the overall differences in stenosis severity, PAV, NCPV, and CPV were statistically significant between perfusion categories (*Table 3*). However, the distributions of these parameters were substantially overlapping between the perfusion categories, rendering most stepwise comparisons statistically non-significant.

The presence of low attenuation plaque increased progressively with ischaemia severity at both the patient level and within each coronary artery. Patient-level prevalence rose from 28% in those with normal perfusion to 60% in those with severe ischaemia (*P* < 0.001). Among individual vessels, low attenuation plaque was most frequently observed in the LAD. RI also increased with ischaemia severity, from a median of 1.4 (1.2–1.5) in patients with normal perfusion to 1.5 (1.4–1.7) in those with severe ischaemia (*P* < 0.001). This pattern was consistently observed across all three vessels, with the LAD showing significantly higher RI compared to LCX and RCA in several perfusion categories.

### The relationship between stenosis degree, plaque features, and ischaemia severity

*Table 4* shows the results of the ordinal logistic regression analyses.

**Table 4:** Ordinal logistic regression analysis of AI-QCT variables associated with ischaemia severity (categorized as normal perfusion, mild ischaemia, moderate ischaemia and severe ischaemia)

	Model 1				Model 2			
	Odds ratio	95% CI	*P*-value	VIF	Odds ratio	95% CI	*P*-value	VIF
**LAD**								
Diameter stenosis, %	1.03	1.02–1.04	**<0**.**001**	1.84	1.03	1.02–1.04	**<0**.**001**	1.98
PAV, %	1.03	1.01–1.05	**<0**.**001**	2.14				
Percent NCPV, %					1.04	1.01–1.08	**0**.**014**	2.19
Percent CPV, %					1.03	1.01–1.05	**0**.**015**	1.47
Low attenuation plaque present	1.24	0.89–1.74	0.204	1.23	1.19	0.83–1.70	0.354	1.40
Remodeling index	1.10	1.01–1.20	**0**.**023**	1.45	1.10	1.00–1.19	**0**.**039**	1.50
**LCX**								
Diameter stenosis, %	1.03	1.02–1.04	**<0**.**001**	1.97	1.02	1.01–1.04	**<0**.**001**	2.07
PAV, %	1.03	1.01–1.05	**0**.**002**	2.36				
Percent NCPV, %					1.05	1.01–1.09	**0**.**011**	2.34
Percent CPV, %					1.02	0.99–1.05	0.121	1.62
Low attenuation plaque present	0.98	0.63–1.53	0.944	1.14	0.93	0.59–1.47	0.761	1.20
Remodeling index	1.02	0.93–1.12	0.712	1.74	1.02	0.93–1.11	0.752	1.75
**RCA**								
Diameter stenosis, %	1.03	1.02–1.03	**<0**.**001**	2.04	1.03	1.02–1.04	**<0**.**001**	2.21
PAV, %	1.04	1.02–1.06	**<0**.**001**	2.46				
Percent NCPV, %					1.04	1.00–1.08	**0**.**031**	2.87
Percent CPV, %					1.04	1.02–1.07	**0**.**002**	1.49
Low attenuation plaque present	1.28	0.84–1.94	0.249	1.26	1.27	0.82–1.99	0.288	1.40
Remodeling index	0.92	0.81–1.04	0.176	1.95	0.92	0.81–1.04	0.177	1.97
**Patient level**								
Diameter stenosis, %	1.04	1.03–1.05	**<0**.**001**	1.84	1.04	1.03–1.05	**<0**.**001**	1.98
PAV, %	1.04	1.02–1.06	**<0**.**001**	2.14				
Percent NCPV, %					1.07	1.02–1.11	**0**.**002**	2.19
Percent CPV, %					1.02	1.00–1.05	0.115	1.47
Low attenuation plaque present	1.17	0.85–1.60	0.330	1.23	1.06	0.76–1.49	0.718	1.40
Remodeling index	1.08	0.99–1.17	0.099	1.45	1.07	0.98–1.17	0.113	1.50

Model 1 included percentages of total plaque (PAV%) whereas Model 2 included percentages of subcomponents of plaque (NCPV% and CPV%). Statistically significant values (*P* < 0.05) are shown in bold.

AI-QCT, artificial intelligence-guided quantitative computed tomography; CI, confidence interval; CPV, calcified plaque volume; LAD, left descending artery; LCX, left circumflex artery; NCPV, non-calcified plaque volume; PAV, percent atheroma volume; RCA, right coronary artery; VIF, variance inflation factor.

In Model 1, diameter stenosis and PAV were significant independent predictors of ischaemia severity both at per patient level and in all the three major coronary arteries. RI was significant only for the LAD.

In Model 2, diameter stenosis and percent NCPV were significant independent predictors of ischaemia severity both at per patient level and in all the three major coronary arteries, whereas RI was significant only in the LAD. Percent CPV was significant independent predictor in the LAD and the RCA but not in the LCx.

The presence of low attenuation plaque was not a significant independent predictor of ischaemia severity in either of the models.

## Discussion

In this relatively large cohort of symptomatic patients evaluated with hybrid coronary CTA and PET MPI, we examined the relationship between quantitative measures of coronary plaque burden and the severity of myocardial ischaemia. Our analysis focused on the major coronary arteries (LAD, LCX, and RCA) and stratified myocardial perfusion into four categories based on regional MBF.

The main findings of our study are four-fold. First, as expected, both vessel luminal narrowing and plaque burden increase with increasing severity of ischaemia, confirming a direct relationship between anatomical disease severity and functional impairment. This is well in line with earlier studies showing that coronary CTA-derived plaque characteristics are strongly associated with myocardial ischaemia.^6–8^ However, where earlier studies have focused on the presence or absence of myocardial ischaemia, our study is novel in relating the AI-QCT-derived coronary plaque measures with the severity of ischaemia, determined by quantitative PET perfusion imaging.

Second, percent non-calcified and calcified plaque volumes were both independent predictors of ischaemia severity for the LAD and RCA, while only non-calcified plaque volume was associated with ischaemia severity in the LCx. This finding might reflect regional heterogeneity in plaque composition and its ischaemic impact although the absence of an association for calcified plaque in the LCx could also be due to statistical variability related to sample size. In our earlier analysis,^11^ similar to the present, percent NCPV was associated with ischaemia in all three vessels. In that analysis, however, percent CPV was independently associated with ischaemia only in the RCA, whereas the findings of the present support association of calcified plaques and ischaemia severity also in the LAD. Our earlier analysis focused on the presence of ischaemia as a binary outcome, whereas the current study assesses ischaemia severity on a graded scale. This may explain why percent CPV now shows a stronger association with LAD ischaemia, as the more granular outcome allows detection of subtler relationships between plaque type and functional impact. In a smaller cohort Liga *et al.*^17^ identified coronary calcium burden and significant stenosis as independent predictors of impaired myocardial flow reserve (MFR), whereas the fibroadipose component was relevant only in stenosed vessels. In our substantially larger cohort using AI-based quantitative plaque analysis of coronary CTA, we confirm the link between plaque burden and perfusion but extend these findings by showing that non-calcified and total plaque volumes—along with calcified plaque in specific territories—are independently associated with increasing ischaemia severity. Differences in imaging methodology, patient sample, and outcome definition (graded ischaemia vs. dichotomous MFR) may explain the broader range of plaque–perfusion associations observed in the present study.

Third, and notably, we observed substantial overlap in the distributions of diameter stenosis, PAV, NCPV, and CPV across the perfusion categories, especially for the LCx and RCA. This overlap suggests that anatomical measures alone may be insufficient to discriminate the severity of ischaemia, and also between normal perfusion and ischaemia, particularly for the LCx and RCA. It highlights the multifactorial nature of ischaemia, which may arise not just from focal luminal obstruction but also from diffuse atherosclerosis, microvascular dysfunction, or adverse plaque morphology (e.g. inflammation, positive remodeling). These findings are consistent with the prior studies showing limited correlation between angiographic stenosis severity and functional ischaemia.^18–20^

Fourth, in this study, we observed important vessel-specific differences in how anatomical plaque metrics correlate with increasing severity of myocardial ischaemia. The LAD consistently demonstrated the highest plaque burden and stenosis severity across all perfusion categories, well in line with earlier studies^9,11^ and reinforcing its central role in the pathophysiology of ischaemic heart disease. In contrast, the LCx showed less pronounced and non-significant pairwise differences across perfusion categories, despite overall trends towards increasing plaque burden. This may reflect diffuse disease or underlying microvascular dysfunction. The RCA demonstrated intermediate patterns, with numerical increases in plaque burden and stenosis but fewer statistically distinct transitions. Notably, CPV increased with ischaemia severity in all vessels, but lacked consistent statistical significance in pairwise comparisons, particularly in the LCx and RCA, suggesting that calcified plaque may be less predictive of functional impairment than non-calcified components. These findings reinforce the importance of regional assessment and vessel-specific interpretation when evaluating plaque-imaging metrics in relation to ischaemia.

The findings from ordinal logistic regression further reinforce these observations. Diameter stenosis and plaque burden were robust independent predictors of ischaemia severity across all territories. However, neither presence of low attenuation plaque—a marker of high-risk plaque—nor RIs were associated with ischaemia severity in this cohort, possibly due to their primary role in plaque vulnerability rather than in coronary flow restriction.

Taken together, our results underscore the value of comprehensive AI-QCT plaque assessment in conjunction with physiological testing. While AI-QCT provides detailed and reproducible quantification of plaque distribution and composition, our findings support the clinical need to integrate anatomical and functional data to fully capture the ischaemic burden and guide management.

### Limitations

This study has some limitations. Only patients with visually suspected obstructive disease on CTA were referred for downstream PET imaging, introducing potential selection bias and possibly underrepresenting patients with non-obstructive or microvascular disease. However, wide range of stenosis and plaque burden are still represented in our cohort as also non-obstructive ‘bystander’ vessels were included in analyses. Despite standard anatomical assumptions in segment-to-vessel assignment and with correction for coronary tree dominance, individual variability in coronary arteries and myocardial perfusion territories may have led to misclassification.

## Conclusions

In symptomatic patients evaluated with coronary CTA and PET MPI, both plaque burden and stenosis severity were associated with increasing severity of myocardial ischaemia. However, substantial overlap in anatomical measures across the severity of myocardial ischaemia suggests that anatomical coronary plaque burden alone does not fully account for functional impairment. These findings highlight the importance of combining quantitative plaque analysis with functional assessment to improve phenotyping of CAD.

## Data Availability

The data underlying this article cannot be shared publicly due to the privacy of individuals that participated in the study.

## Lead Author Biography

**Tanja Kero, MD, PhD**, is a physician and researcher in Nuclear Medicine and PET at the Department of Surgical Sciences, Uppsala University, Sweden. Her research focuses on the development and clinical application of advanced molecular imaging techniques, with a particular emphasis on nuclear cardiology and positron emission tomography (PET). Dr. Kero’s work aims to improve diagnostic accuracy and patient management in cardiovascular disease through innovative nuclear imaging methods and interdisciplinary collaboration.
